# Extracorporeal Membrane Oxygenation in Severe Acute Respiratory Distress Syndrome Caused by *Chlamydia psittaci*: A Case Report and Review of the Literature

**DOI:** 10.3389/fmed.2021.731047

**Published:** 2021-10-15

**Authors:** Lu Wang, Zhaokun Shi, Wei Chen, Xianjin Du, Liying Zhan

**Affiliations:** Department of Critical Care Medicine, Renmin Hospital of Wuhan University, Wuhan, China

**Keywords:** *Chlamydia psittaci* (*C. psittaci*), pneumonia, extracorporeal membrane oxygenation (ECMO), next-generation sequencing (NGS), infection

## Abstract

**Background:** Infection of *Chlamydia psittaci* (*C. psittaci*) could lead to serious clinical manifestations in humans, including severe pneumonia with rapid progression, adult respiratory distress syndrome (ARDS), sepsis, multiple organ dysfunction syndromes (MODS), and probably death. Implementation of extracorporeal membrane oxygenation (ECMO) in the patient with severe ARDS gives a promising new method for recovery.

**Case Presentation:** We report our successful use of venovenous (VV) ECMO in a 48-year-old man who manifested with severe respiratory distress syndrome, acute kidney injury, and septic shock caused by a diagnosis of pneumonia. After the combination of therapy including anti-infection, mechanical ventilation, and continuous renal replacement therapy (CRRT), acute inflammatory syndrome developed. However, his respiratory status rapidly deteriorated. Then, venoarterial (VA)-ECMO support was placed on the patient as suddenly slowing of the heart rate. Harlequin (North-South) syndrome occurred after ECMO initiation. A series of the process could not relieve hypoxia in the upper body. At last, transition to VV-ECMO improved hypoxia. The duration of VV-ECMO was 7 days and the mechanical ventilation was weaned on the next day. On the day of ECMO weaning, nanopore targeted sequencing (NTS) of bronchoalveolar lavage fluid (BALF) reported the presence of *C. psittaci*. After 19 days of critical systemic rehabilitation and combination therapy, the patient fully recovered from *C. psittaci*.

**Conclusion:** This is the first reported case of the patient receiving ECMO for *C. psittaci* pneumonia. ECMO puts the lungs on temporary rest, promotes the recovery of pulmonary function, and also wins time for finding the pathogens, which is crucial in the treatment of rare pathogens.

## Background

*Chlamydia psittaci* is a type of bacteria that often infects birds. The bacteria can also infect people exposed to the infected birds and cause a disease called psittacosis. Those who have contact with pet birds and poultry, including the people who work in bird-related occupations, are at an increased risk of infection. In 2018, a multistate psittacosis outbreak occurred among the poultry workers that had 13 laboratory confirmed cases (1). A study found that out of the 311 parrots examined in China, 35.37% were seropositive and species, gender, age, season, and geographical location were identified as the risk factors (2). The occurrence of *C. psittaci* genotype A in the droppings of the two pet parrots in China suggests potential environmental contamination with *C. psittaci* and may raise public health concerns (2). A few isolated cases or outbreaks of psittacosis have been reported, usually presenting with severe pneumonia.

Extracorporeal membrane oxygenation (ECMO) is used to treat patients with severe, life-threatening conditions of the heart and lungs, but some diseases may lead to progressive organ dysfunction such as liver failure or severe neurologic injury. Then, these conditions with a poor prognosis may warrant a discussion about discontinuing ECMO support (3). An ECMO study in the United Kingdom found that 95% of the patients with severe respiratory failure had venovenous (VV) ECMO alone (4). The survival rate at ECMO intensive care unit (ICU) discharge was 74% including 71% for the patients with respiratory failure (4). VV-ECMO support is an established treatment of acute respiratory distress syndrome (ARDS) and enables to minimize ventilator-induced lung injury (VILI) as rescue therapy in ARDS patients (5, 6). But, the successful use of ECMO to treat patients with psittacosis has not been published yet.

Therefore, the use of ECMO in patients with psittacosis needs further study and discussion. However, the timing of ECMO initiation, ECMO model [VV, venoarterial (VA) or the other types], and length of ECMO also need to be studied.

## Case Presentation

**Figure 1:**
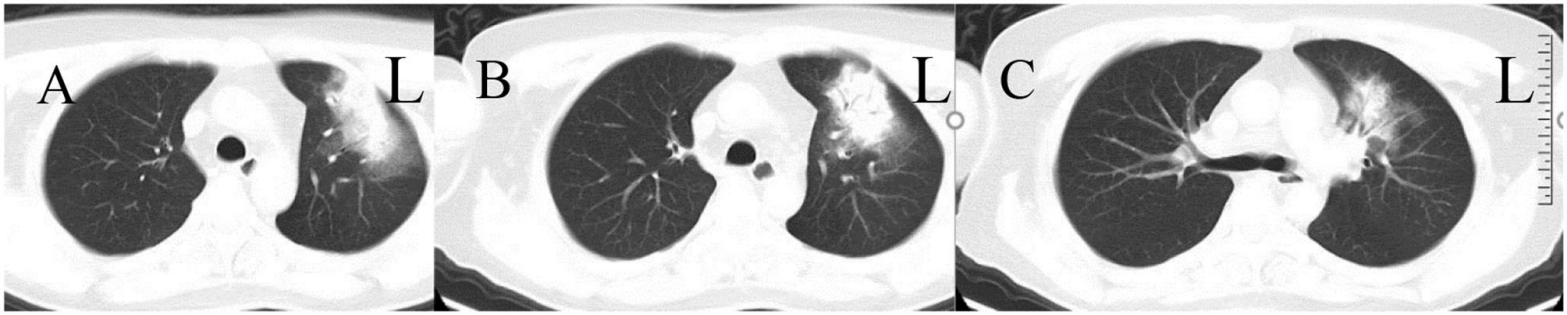
CT scan of the patient (**A–C** show CT scans on the 0th day after admission).

This was a case of a 48-year-old community inspector with a history of possible hemorrhoids (hematochezia) for 2 months, gastric ulcer, cervical spondylosis, and penicillin allergy, who presented in January 2021 with fever, diarrhea, chest pain, fatigue, and syncope. The patient, who lived in a central urban area of Wuhan, China, had dizziness for 3 days and fell three times before being admitted to the hospital. Fever, diarrhea, and chest pain appeared 1 day before with chest pain subsided 5 min later. Physical examination: Temperature was 39.6°C, heart rate was 126 beats/min, breath rate was 20 breaths/min, blood pressure was 148/77 mm Hg, oxygen saturation (SpO_2_) was 98%, and lung auscultation was clear. Other physical examinations were negative. CT scan of the chest showed left upper zone consolidation (Figure 1). Blood tests indicated a white blood cell count 2,910/mm^3^, neutrophil count 2,630/mm^3^, lymphocyte count 100/mm^3^, hemoglobin 82 g/l, platelet count 7,300/mm^3^, C-reactive protein 193.9 mg/l (normal value <10 mg/l), serum amyloid A (SAA) protein > 300 mg/l (normal value <10 mg/l), and procalcitonin (PCT) 6.51 ng/ml. Galactomannan enzyme immunoassay (GM-EIA) shows 0.12 (normal value 0–0.49) and fungus (1 → 3)-β-D-glucan test shows <37.50 pg/ml (normal value <70 pg/ml). Blood and sputum culture were performed. The patient was diagnosed with community-acquired pneumonia and empirical intravenous antibiotic therapy was started with ceftazidime. 1 day later, he fainted suddenly with SpO_2_ 80–90%, partial pressure of oxygen (pO_2_) 71 mm Hg, and then transferred to the ICU due to deterioration of respiratory status, severe desaturation, and delirium. At the same time, wet and dry rales could be heard in both the lungs, and the Acute Physiology and Chronic Health Evaluation II (APACHE II) and the Sequential Organ Failure Assessment (SOFA) scores were 18 and 8, respectively (Table 1). Tracheal intubation and mechanical ventilation were performed with positive end-expiratory pressure (PEEP) 10 mm Hg and a fraction of inspired oxygen (FiO_2_) 100%. Flexible bronchoscopy was performed and showed pulmonary edema without the other abnormalities. To get more information on etiology, bronchoalveolar lavage fluid (BALF) samples were obtained for nanopore targeted sequencing (NTS) and sputum culture. The results of echocardiography showed the size of the ventricles and atria were normal, the thickness and amplitude of movement of the ventricular septum and the free wall of the left ventricle, the morphology and activity of the valves, and the opening and closing of the valves were normal. Simultaneously, antibiotic therapy was changed to meropenem and ganciclovir. In the following days, he presented with progressive worsening of the bilateral pulmonary infiltrates, high ventilatory parameters, and increasing serum PCT and creatinine levels with the signs of septic shock. Ultrafiltration was required for oligoanuria and severe fluid was overload. On the fourth day after intubation, pO_2_/FiO_2_ was reduced to 59 and bronchoscopy showed severe airway mucosal edema. Then, ECMO was commenced on day 5 in the ICU. VV-ECMO was planned originally; VA-ECMO with the initial flow set at 4 L/min was finally performed as the heart rate of the patient sharply dropped before the inserting tubes. Then, the patient experienced intermittent episodes of significant hypoxemia with SpO_2_ ranging from 41 to 81% corresponding to right radial artery partial pressure of arterial oxygen (PaO_2_) ranging from 49 to 58 mm Hg. To find out the reason for hypoxemia, we raised the flow rate of ECMO from 4.0 to 4.5 L/min. This adjustment could not eliminate hypoxemia. The PaO_2_ of the right radial artery was 60 mm Hg, while the PaO_2_ of the right dorsal foot artery was 400 mm Hg. A severe North-South syndrome was presented as differential cyanosis. This cannot be eliminated by venoarterial venous (VAV)-ECMO with the presence of an internal jugular venous shunt of 18 Fr arterial cannulas. After the confirmation of ultrasound, the function of the heart was normal and at last, we converted to VV-ECMO. On day 2 (day 6) of ECMO, nucleic acid screening of the pharyngeal swab showed chlamydia. Antibiotic therapy with azithromycin was administered. Methylprednisolone pulses and respiratory physiotherapy were implemented. Improvement of pulmonary infiltrates was observed in the following days. After 7 days, ECMO support was discontinued (day 12) and ventilation was converted to high flow humidification oxygen therapy on the next day (day 13). On the 13th day after admission, the NTS results from the third sample of BALF showed the presence of *C. psittaci* and the readings of the sequences were 96. Therefore, the antibiotics were changed to meropenem and doxycycline accordingly. Signs of infection were improved after combined dialysis, nutrition, and other treatments. X-ray and CT scan of the chest demonstrated improved diffuse ground-glass infiltrates. Multiple cultures of blood and BALF were negative. The patient was weaned off the ventilator on the 17th day after the admission. Throughout the course of treatment, the indicators of infection and renal function tended to improve (Table 2). He regained some kind of consciousness, but could not give the exact answer and the vasoactive drugs were significantly downregulated.

**Table 1:** Characteristics of the patient and the methods of treatment.

	**Feature**
Age, yr	48
Sex	Male
Clinical features	Fever, diarrhea, chest pain, fatigue, and syncope
Smoke	No
Drink	No
Surgery	No
Hormone/immunosuppressive therapy	No
Chronic diseases	
Hypertension	No
Diabetes	No
Kidney failure	No
Chronic bronchitis	No
Treatment	
Vasoactive drugs	Yes
Arterial puncture	Yes
ECMO	Yes
Tracheotomy	Yes
CVC	Yes
Nasogastric tube	Yes
Urinary catheters	Yes
CRRT	Yes
SARS score	56
APACHE II	18
SOFA	8
Mechanical ventilation duration(days)	16

*CVC, central venous catheter; ECMO, extracorporeal membrane oxygenation; CRRT, continuous renal replacement therapy*.

**Table 2:** Ventilator and the ECMO parameters, indicators of infection, and renal function.

	**D1**	**D2**	**D3**	**D5**	**D7**	**D8**	**D9**	**D12**	**D17**
FiO_2_	100%	60%	60%	100%	100%	40%	40%	35%	35%
PEEP (cmH_2_O)	10–8	8	8–5	8–10	8–10	5	5	5	3–5
Pplat	18	15	16	18	18	15	14	12	8
CL (ml/mmHg)	35	38	42	33	38	41	49	56	65
Vt (ml)	300–400	400–450	400–450	300	300–400	300–400	400–45–	400–500	400–500
PCO_2_ (mmHg)	30	32	42	42	42	55	36	38	34
PO_2_ (mmHg)	71	97	167	59	439	96	102	71	80
P/F ratio (mmHg)	71	161	228	59	439	192	240	177.5	228.6
ECMOration rate (revolutions/min)	–	–	–	3,000	3,200	3,200	3,200	2,200	–
ECMO flux (3 L/min)	–	–	–	3.3	3.3	3.3	3.3	1.4	–
ECMO FiO_2_	–	–	–	100%	100%	100%	100%	40%	–
Dosage of vasoactive drugs	NE 20 ug/min	NE 15 ug/min	NE 15 ug/min	NE 30 ug/min pavee DFA 55 ug/min	NE 15 ug/min pavee DFA 30 ug/min	NE 15 ug/min pavee DFA 30 ug/min	NE 15 ug/min pavee DFA 30 ug/min	–	–
Antibiotics	Meropenem and ganciclovir	Meropenem and ganciclovir	Meropenem and ganciclovir	Meropenem and ganciclovir	Meropenem, ganciclovir and azithromycin	Meropenem, ganciclovir and azithromycin	Meropenem, ganciclovir and azithromycin	Meropenem and doxycycline	Doxycycline
W.B.C (mm^3^)	3,830	6,090	10,950	13,290	6,270	7,820	7,650	5,760	2,770
PCT (ng/ml)	6.51	60.1	58.7	35.9	16	9.23	5.2	1.38	0.84
Cr (umol/L)	178	243	180	242	180	139	199	171	209
Urine volume (ml)	1,450	2,060	1,400	300	70	50	30	40	1,700

*FiO_2_, fraction of inspired oxygen; PEEP, positive end-expiratory pressure; VT, tidal volume; Pplat, platform pressure; CL, lung compliance; pCO_2_, partial pressure of carbon dioxide; pO_2_, partial pressure of oxygen; WBC, white blood cell; PCT, procalcitonin*.

## Discussion

*Chlamydia psittaci* is a gram-negative, obligate intracellular parasite that is mainly transmitted to humans through contact with the infected birds (such as parrots and poultry), inhalation of aerosols, feces, or feather dust from the nasal secretions of the infected birds. The disease caused by *C. psittaci* infection is called psittacosis, which is considered a rare cause of pneumonia. A meta-analysis of an observational study found that about 1% of the community-acquired pneumonia was caused by *C. psittaci* (7). Probably due to the lack of routine detection of *C. psittaci* and the sensitivity and specificity of the common diagnostic methods, it is difficult to determine the accurate incidence and prevalence of *C. psittaci* (8, 9). Contact with the birds or poultry is the main risk factor for psittacosis. It has been reported that among 1,136 patients, 72% of the patients had pets or had contact with birds or poultry in a domestic environment and 6% had exposure to wild birds, 12% are poultry workers, and only 10% have no relevant contact history (10, 11). In addition to the parrots, poultry is also an important source of infection of *C. psittaci*. A study found that the seropositivity rates of *C. psittaci* in the chickens, ducks, and pigeons sold in Northwest China were 13.3, 38.9, and 31.1%, respectively (12).

In this case study, a positive environmental link has been identified. During the same period, the Chinese news outlets reported psittacosis cases in Changsha, Hunan province, and Zhongshan, Guangdong province. Although this case lived in an urban area, there were large forests and birds in the community. Therefore, the patients with a history of exposure to the birds or poultry should be alert to the possibility of atypical infection of the pathogen, especially *C. psittaci*. The incubation period ranges between 5 and 14 days. In a study of 135 patients, all the patients had a fever as the main manifestation and 61% of patients were accompanied by chills. Although 82% of the patients complained of cough, it often appeared later (13). Its typical clinical manifestations are fever, chills, headache, cough without sputum, and gastrointestinal symptoms. In severe cases, severe pneumonia, endocarditis, jaundice, and neurological complications may be developed. Since *C. psittaci* is difficult and dangerous to culture and is highly infectious, serological examination is usually used for the diagnosis. Among them, the most sensitive and specific is a microimmunofluorescence assay showing antibody titers at least 4-fold higher than the upper limit of normal in the duplicate serum samples or the titer of the IgM antibody is ≥1:16. The development of the PCR has greatly simplified DNA analysis and shortened the laboratory time to detect *C. psittaci*. This method can quickly and specifically identify the pathogens and perform genotyping at the same time, but many authors described the assays that were less sensitive and detected only when the high loads were observed during the acute phase of the disease. Nowadays, the microbiology laboratories do not routinely screen the above tests, but only in the specialized laboratories, so it is difficult for clinicians to diagnose psittacosis. Through the development of the second-generation metagenomic intervention technology [metagenomic next-generation sequencing (mNGS)], a variety of the pathogens in the different specimens can be quickly and accurately identified including atypical pathogens, viruses, and fungi that are difficult to cultivate. Thus, mNGS, also known as deep sequencing, is widely favored because of its rapid detection. This technology is based on collecting the human genes followed by the amplification and sequencing of pathogen nucleic acids and then high-throughput sequencing, machine-learning algorithms, and bioinformatics pipelines were performed. Its versatile detection has an advantage for the diagnosis of rare pathogenic bacteria in difficult cases. mNGS technology was done to diagnose *Chlamydia pneumoniae* by the BALF samples when the situation deteriorated in our procedure. With rapid analysis, this approach can quickly detect most of the pathogens and overcoming conventional culture-independent evaluation of the little clinical samples (microliter volumes) or prior antibiotic exposure. mNGS also provides additional data including bacterial DNA burden and community diversity that may help to identify pathogenic bacteria. New technology for performing targeted screening of NTS is a type of mNGS (14); therefore, it was developed to detect *C. psittaci*. Studies have shown that NTS is sensitive for detecting respiratory viruses (15), but its use in the detection of *C. psittaci* is less reported, so its rapid detection could be very beneficial for the early diagnosis and treatment of patients with parrot fever. In this case study, three BALF samples were sent to analyze by NTS technology. Only the last one reported *C. psittaci*. Nevertheless, we only found *C. psittaci* in the third test of NTS after we deeply discussed this case with the laboratory staff and the readings of the sequences were extremely low that is easy to ignore. In consistent with previously reported (16), the difference between blood and BALF may be due to the fewer effective cells in the BALF. Therefore, to obtain a meaningful diagnosis, the doctors need to consider the analysis of NTS combined with the clinical manifestations. NTS also has other disadvantages that limit its public application such as without drug sensitivity, the cost, and unavailable in mostly small hospitals, which increases the possibility of missed or misdiagnosis of the rare pathogenic bacteria in difficult cases.

*Chlamydia psittaci* belongs to the Chlamydia family (13) and tetracyclines, macrolides, and quinolones which suppress DNA and protein synthesis could be the appropriate antibacterial drugs (17). The first-line medication is tetracycline drugs for at least 3 weeks in order to avoid recurrence. If tetracyclines are restricted in some patients, such as children, pregnant women, or allergies, macrolides can be selected as an alternative treatment. In some cases, quinolones are effective but are less effective compared to tetracyclines and macrolides (18). The patient was presented in this study had used azithromycin and meropenem according to the nucleic acid test of pharyngeal swab that showed *Chlamydia*, but the patient still had fever with pulmonary function deterioration, which means poor efficacy or insufficient treatment. These two drugs are supposed to be second-line treatment and in effect (19, 20), but, at first, the condition of the patient progresses too fast and the disease is too severe. After NTS of BALF reported *C. psittaci*, timely adjustment of the antibacterial treatment based on doxycycline minimized the prognosis time and disease process. At the same time, NTS testing did not report other pathogens, thereby reduced the use of the unnecessary antibacterial drugs, effectively reduced the hospitalization costs, and avoided the emergence of the drug-resistant bacteria.

**Figure 2:**
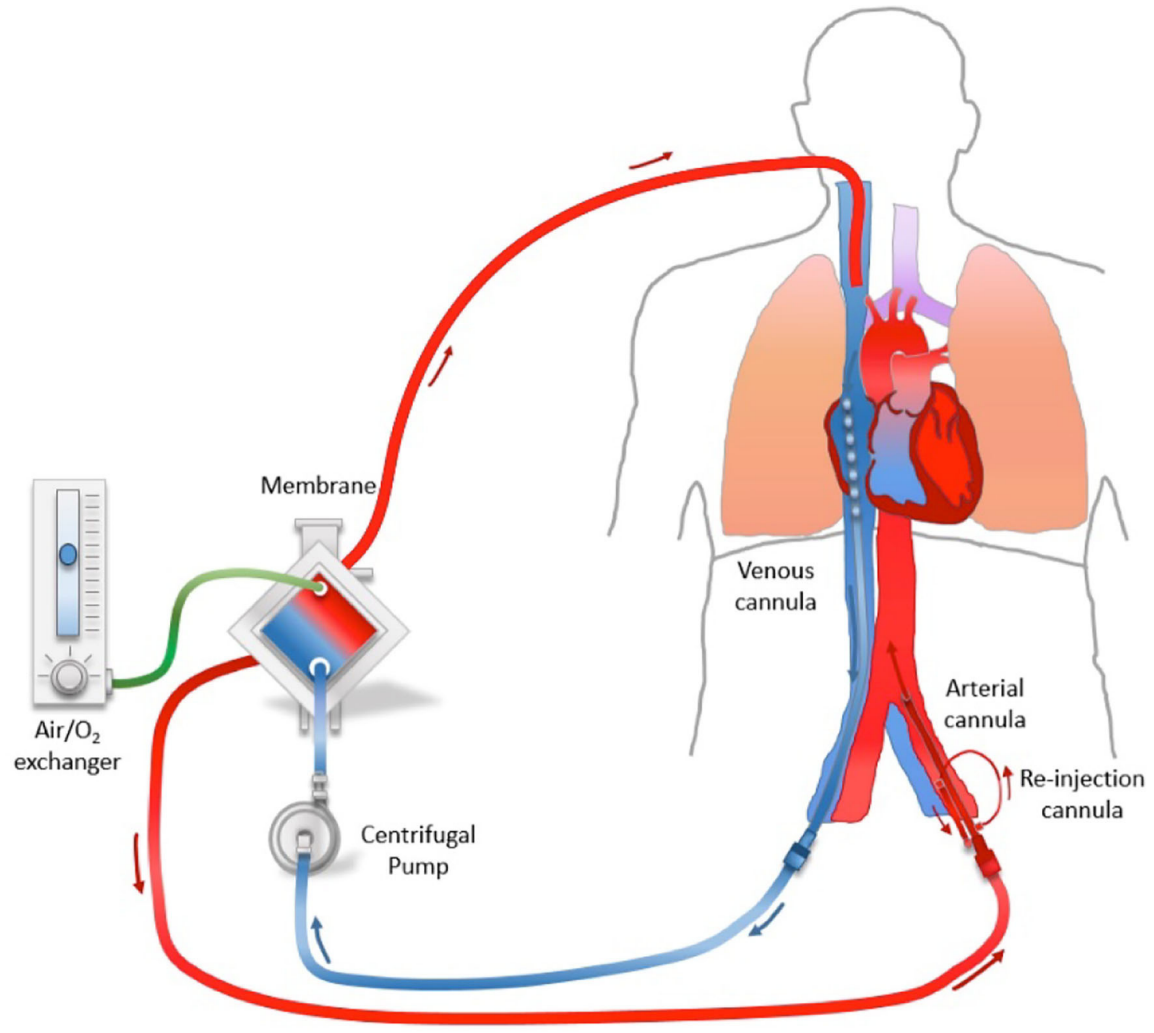
North–South syndrome.

In the case of ventilator treatment, the condition of the patient deteriorated on the 5th day and the adjustment of ventilator parameters could not meet the needs of the patient, so ECMO treatment was given. In ECMO, blood is pumped outside of the body to a heart-lung machine, which removes carbon dioxide and fills oxygen into the blood back to the tissues in the body, which could be an effective technology to improve organ oxygenation when positive-pressure ventilation and prone position are inadequate to improve the blood oxygen saturation. There are two types of ECMO. The VA-ECMO is connected to both a vein and an artery and is used when there are problems with both the heart and lungs. In most cases of ECMO for severe acute respiratory failure, VV-ECMO is selected from which blood is pumped and returned to a central vein. ECMO allowed further reduction in ventilation-induced lung damage and the search of a diagnostic procession that included culture and mNGS making the patient to the definitive diagnosis, specific antibiotics, immunosuppressive treatment, and recovery. In the present case study, we first described the patient with psittacosis complicated by refractory respiratory failure and rescued with VV-ECMO. In this case study, VA-ECMO cannulation was selected because of the risk of hemodynamic collapse after a sharp reduction of heart rate. This choice leads to the North-South syndrome soon afterward, which is a form of Harlequin syndrome. North-South syndrome occurs in 38% of ECMO operations (21). Some scientists argue that draining from the superior vena cava (SVC) via a multistage cannula inserted in the right internal jugular vein is neutralized (22), but drainage of the internal jugular vein (also called VAV-ECMO) seems do not solve the problem in this case. In the VAV-ECMO circuit, with drainage from a cannula inserted in the femoral artery and then return of a cannula inserted in the internal jugular vein (tip in the SVC next to the right atrium) and the femoral vein (tip in the inferior vena cava), the blood volume of the upper body reduced with insufficient supply of oxygen and low blood pressure. It is also indicated that the patient had a severe pulmonary infection and poor oxygenation (Figure 2).

## Conclusion

In summary, this is the first case of pulmonary infection with ECMO occurring in a patient in China suggesting that ECMO plays an increasing role as an opportunistic pathogen in immunocompromised patients. With the consent of the patient (supply figure), this case provides a reminder that the clinicians should expect the unexpected in terms of the infectious agents and emphasizes the importance of the microbiological diagnostic procedures.

## Data Availability Statement

The original contributions presented in the study are included in the article/supplementary material, further inquiries can be directed to the corresponding author/s.

## Ethics Statement

Written informed consent was obtained from the individual(s) for the publication of any potentially identifiable images or data included in this article.

## Author Contributions

All authors listed have made a substantial, direct and intellectual contribution to the work, and approved it for publication.

## Conflict of Interest

The authors declare that the research was conducted in the absence of any commercial or financial relationships that could be construed as a potential conflict of interest.

## Publisher's Note

All claims expressed in this article are solely those of the authors and do not necessarily represent those of their affiliated organizations, or those of the publisher, the editors and the reviewers. Any product that may be evaluated in this article, or claim that may be made by its manufacturer, is not guaranteed or endorsed by the publisher.
